# Association analysis revealed loci linked to post-drought recovery and traits related to persistence of smooth bromegrass (*Bromus inermis*)

**DOI:** 10.1371/journal.pone.0278687

**Published:** 2022-12-07

**Authors:** Fatemeh Saeidnia, Mohammad Mahdi Majidi, Aghafakhr Mirlohi, Benyamin Ahmadi

**Affiliations:** 1 Assistant Professor of Agricultural and Horticultural Science Research Department, Khorasan Razavi Agricultural and Natural Resources Research and Education Center, Agricultural Research, Education and Extension Organization, Mashhad, Iran; 2 Department of Agronomy and Plant Breeding, College of Agriculture, Isfahan University of Technology, Isfahan, Iran; 3 Department of Horticulture, College of Agriculture, Isfahan University of Technology, Isfahan, Iran; KGUT: Graduate University of Advanced Technology, ISLAMIC REPUBLIC OF IRAN

## Abstract

Association analysis has been proven as a powerful tool for the genetic dissection of complex traits. This study was conducted to identify association of recovery, persistence, and summer dormancy with sequence related amplified polymorphism (SRAP) markers in 36 smooth bromegrass genotypes under two moisture conditions and find stable associations. In this study, a diverse panel of polycross-derived progenies of smooth bromegrass was phenotyped under normal and water deficit regimes for three consecutive years. Under water deficit, dry matter yield of cut 1 was approximately reduced by 36, 39, and 37% during 2013, 2014, and 2015, respectively, compared with the normal regime. For dry matter yield of cut 2, these reductions were approximately 38, 60, and 56% in the same three consecutive years relative to normal regime. Moreover, water deficit decreased the RY and PER of the genotypes by 35 and 28%, respectively. Thirty primer combinations were screened by polymerase chain reaction (PCR). From these, 541 polymorphic bands were developed and subjected to association analysis using the mixed linear model (MLM). Population structure analysis identified five main subpopulations possessing significant genetic differences. Association analysis identified 69 and 46 marker-trait associations under normal and water deficit regimes, respectively. Some of these markers were associated with more than one trait; which can be attributed to pleiotropic effects or tightly linked genes affecting several traits. In normal and water-deficit regimes, these markers could potentially be incorporated into marker-assisted selection and targeted trait introgression for the improvement of drought tolerance of smooth bromegrass.

## Introduction

Under the changing climatic context, drought is becoming the most significant and acute problem affecting crop growth, survival, and persistence in many regions of the world, particularly in arid and semi-arid regions [1, 2]. Developing drought-tolerant varieties is an essential objective of plant breeding programs. It is expected to be a key component in strategies to mitigate climate change, minimize losses, and ensure production stability [3]. Given the advantages that perennial forage grasses such as smooth bromegrass have in agriculture and forage sustainability for animal farming, they can be a valuable substitute for annuals under drought conditions. In these species, forage yield, persistence, and recovery are the most important agronomic features in areas with periodic drought in summer [4, 5].

Successful adoption of perennial forage species depends on the persistence of the species, which is mainly defined as ‘plant survival through repeated summer droughts and its ability to produce continuously high forage yields across several growing seasons’ [4, 6]. In other words, perennial grass persistence depends on the plant’s ability to maintain a viable crown at the soil surface from which growth regenerates. When genotypes are spaced planted, the difference in performance throughout the years can give an idea of persistence [7]. In semi-arid rainfed regions, intense summer droughts reduce all production of plants. In these regions, the most relevant criterion for plant tolerance is drought survival, i.e. the ability of plants to remain alive during drought and recover when rehydration occurs [8]. However, it is also known that poor regrowth can result in low persistence [9]. Therefore, during drought, trait selection for high recovering ability may be of more economic significance than selecting for improved growth [10, 11]. This will enable forage plants to persist in swards or pastures and to improve their competition with less drought-tolerant species [12, 13]. Therefore, rapid recovery of damaged plant tissues and the regrowth of new tissues following drought stress are important in perennial grass management [11]. The potential of a plant to recommence growth and grain yield after experiencing water deficit is defined as drought recovery [14]. Successful post-drought recovery is contingent on various mechanisms, such as compensatory growth of remaining tissues, retention of intact growing points throughout water deficit, and mobilization of the organism’s carbohydrate reserves [11].

Besides, plant survival and recovery have been associated with summer dormancy, dehydration tolerance in surviving tissues, extensive root system, and the ability of roots to extract water at low soil water potentials [15, 16]. Summer dormancy is acknowledged as an important drought tolerance trait in cool season perennial grasses that originating in regions that experience protracted summer drought [17]. This trait is an endogenously and temporary suspension of visible growth of any plant structure containing a meristem [15], which leads to the reduction or cessation of leaf growth and possible senescence of herbage expressed under non-limiting irrigation in summer [18]. Shaimi et al. [16] showed that, in orchardgrass, summer dormancy is associated with superior persistence and recovery after severe drought under arid and semi-arid conditions. Nevertheless, complete summer dormancy seems to be associated with low productivity [18].

Drought tolerance is a complex quantitative trait involving diverse and multiple molecular and physiological mechanisms, signal transduction, and metabolic pathways [19]. Therefore, a promising strategy to facilitate selection and breeding for drought tolerance is to use from indirect selection of related traits to drought tolerance (i.e. drought survival, post-drought recovery, and persistence) through identifying inherited genetic markers linked to those traits. The basic prerequisite for marker-assisted selection (MAS) is the availability of markers strictly associated with genes or QTLs which can be used to dissect complex traits [20, 21].

As an essential tool for accelerating the rate of genetic gain, MAS’s first step is dissecting marker-trait associations (MTAs) [22]. Genome-wide association study (GWAS) or association mapping, which is also known as linkage disequilibrium (LD) mapping, has recently been proved to be a valuable and powerful alternative to bi-parental QTL mapping for identifying MTA in plant populations [23, 24]. Multi-parent cross designs bridge the two approaches and dramatically increase mapping resolution and power by incorporating greater genetic diversity [25]. The power of association analysis to identify and characterize loci associated with complex traits is highly affected by admixtures of populations [26]. Therefore, it is necessary to evaluate the population structure to avoid identifying false positive or spurious associations between markers and traits [27]. In addition, utilizing a mixed-model approach involving multiple levels of relatedness is essential in avoiding both types of error (types I and II) [20, 28]. Association analysis can be performed using a general linear model (GLM) and mixed linear model (MLM). In MLM, both the kinship matrix (K) and population structure (Q) are incorporated, whereas, in the GLM, only population structure information is used as a covariate [20].

In recent years, association analysis in forage grasses has been applied extensively for dissecting the genetic bases of different quantitative traits in several species such as perennial ryegrass (*Lolium perenne*) [29–32], tall fescue (*Festuca arundinacea*) [33, 34], and orchardgrass (*Dactylis glomerata* L.) [35–37]. These studies have demonstrated that GWAS is an efficient technique for identifying genomic regions linked to quantitative traits. Moreover, Archangi et al. [38] in a study on *Cuminum cyminum* L. identified complementary DNA-amplified fragment length polymorphism (cDNA-AFLP) markers associated with the plant height and several seed yield-related traits, which some of these markers were significantly associated with more than one trait under two water status conditions. In smooth bromegrass, the use of association analysis in identifying links between genes or markers with complex traits, such as those related to persistence and recovery after the drought is still in its infancy.

Due to the importance of smooth bromegrass, a comprehensive study was started on this species at the Isfahan University of Technology with collecting a wide and diverse germplasm in 2003. The pre-breeding studies were performed on this germplasm and based on the results of these studies, some parents were selected for polycross and used for the production of half-sib (HS) families. After 3 years of field evaluation, single plants were randomly selected within these HS families, making a collection containing a total of 36 genotypes (for instance see [7, 39–44]). The next step after those pre-breeding studies is to evaluate the possibility of using marker-assisted selection for identifying of suitable genotypes for the development of synthetic varieties. On the other hand, this experiment was conducted under two irrigation regimes to identify marker-trait associations and select drought tolerant genotypes suitable for the development of synthetic varieties. Hence, this study aimed to identify genetic loci associated with the productivity, persistence, post-drought recovery, and summer dormancy in normal and water-deficit regimes and find stable marker loci associated with the traits mentioned above.

## Materials and methods

### Plant materials and field evaluations

Plant materials consisted of 216 clones randomly selected from a large nursery with 1800 single spaced-plant polycrossed progenies resulting from 36 parental genotypes of smooth bromegrass (*Bromus inermis*). The parental genotypes (S1 Table) were randomly selected from a diverse and large nursery population (containing about 2000 plants) at Isfahan University of Technology Research Farm in 2006, which mainly consisted of natural ecotypes of smooth bromegrass from wide geographical areas of Iran plus some foreign accessions [44]. Foreign accessions were provided by Hungarian Institute for Agrobotany (HIFA), Tapioszele, Hungary. To select the parental plants, all genotypes in the nursery were subjected to cluster analysis based on several agronomical and morphological data of previous studies. Based on the size of each cluster, a similar proportion of genotypes were randomly selected. The parental genotypes were clonally propagated in a greenhouse, and transferred to an isolated polycross nursery to produce new genetic combinations. The polycross nursery was consisted of a balanced lattice square design with eight replicates. Seeds of each genotype from all of the eight replications were harvested separately and mixed together in an equal proportion, and then a random sample was collected from the bulked seed mixture of each parental genotype to produce half-sib families. Polycross seeds were grown in plastic boxes in a greenhouse. The well-established and uniform seedlings (each containing 4 tillers) were space planted in the field according to a randomized complete block design with four replications. Therefore, a large population containing ~2000 plants (clones) was created which 216 clones were developed as the plant materials for the present study and were subjected to the phenotyping, genotyping, population structure, and association analysis. For this purpose, these plants were space-planted in the field according to a randomized complete block design with 12 replications in March 2012. Six replications were allocated to each of the two moisture regimes (normal and water deficit). Within and between row spacing was 50 cm in each block.

Genotypes were evaluated under normal and water deficit regimes during 2013–2016, in which irrigation occurred when 50% and 85% of the total available soil water was depleted from the root zone, respectively, following accepted methods of determination of evapotranspiration [45]. Water deficit was continuously applied in each year of the experiment from early May to early October (plant growing period). Depending on the weather conditions, the irrigation intervals were variable during the growing season and between the two moisture regimes. To determine the gravimetric soil–water content and the irrigation times, soil samples were taken daily from different sites of each moisture regime before irrigation at depths of 0–20, 20–40, and 40–60 cm, using a hand auger [46]. The irrigation depth was calculated as follows (Eq 1):

$$I=[(FC‐G_{irr})/100]\;D\times B$$ (1)

where I is the irrigation depth (cm), FC is the soil gravimetric moisture percentage at the field capacity, G_irr_ is the soil gravimetric moisture percentage at the time of irrigation, D is the root-zone depth, and B is the soil bulk density at root-zone (1.4 g cm^-3^). A basin irrigation system was used for watering. In this system, water was delivered to the field via a pump station and polyethylene pipes. A volumetric counter was used to measure water volume applied under each moisture regime.

### Phenotyping

During the plant establishment year, no data were recorded. Traits were measured for three years during the growing seasons of 2013 to 2015 (S2 Table). In these years, when flowering in all plots was completed (about early summer), the aboveground biomass of each plant was harvested manually from 5 cm aboveground, dried at 75°C for 48 h, and recorded as dry matter yield weight per plant. In each year of the experiment, two harvests of above-ground biomass were undertaken. The first harvest (spring forage yield; DMY1) was done after pollination assessment in late spring (10 June), and the second (summer forage yield; DMY2) in late summer (5 September) to assess complete growth. In order to evaluate the seasonal growth activity, summer dormancy index (SDI) was calculated as the ratio of the SUFY of a genotype to the SPFY of the same genotype as follows (Eq 2) [18]:

SDI={100−[(summerforageyield/springforageyield)×100]}/10 (2)

After three years of field evaluation, all genotypes were assessed for post-drought recovery in the field in 2016. For this purpose, after the first harvest in June 2016, severe water deficit was imposed on both previous moisture regimes (normal and water deficit) by ceasing irrigation for 60 days (from 1st June to 31st July) until complete desiccation of the grass foliage. All plants were subsequently irrigated to the point of field capacity every week to allow water deficit recovery. After six weeks of regular re-watering (15 September), traits related to recovery were measured. Recovery yield (RY) was obtained by measuring the above-ground biomass of each genotype after withholding irrigation and re-watering. The degree of recovery after drought (DRAD) was visually scored based on a scale of 0 to 9. In this respect, green and fully hydrated leaves were rated as 9, and desiccated brown/dead leaves were graded as zero. Persistence (PER) of genotypes was calculated as the difference in dry matter yield of the first cut in the fourth year (2016) from the dry matter yield of the first cut in the second year (2014) (Eq 3) [7]:

PER=DMY1(2016)‐DMY1(2014) (3)

### Genotyping

Young leaf tissues of smooth bromegrass plants were used to extract genomic DNA using the modified method described by Murray and Thompson [47]. The quality and quantity of DNA were determined by electrophoresis in 1% agarose gel. Genotyping using sequence related amplified polymorphism (SRAP) markers was performed following the method of Li and Quiros [48]. Among the SRAP markers available, 30 primer combinations were screened by polymerase chain reaction (PCR). PCR reactions were conducted in a final volume of 10 μL consisting of 1.5 μL of DNA, 1 μL of forward primer, 1 μL of reverse primer, 5 μL of master mix (Amplicon) and 1.5 μL of distilled water, using a BIO-RAD thermocycler. For SRAP analysis, samples were subjected to the following thermal profile: the five cycles including initial denaturation of 1 min at 94˚C, annealing of 1 min at 35˚C, and extension of 1 min at 72˚C, followed by 35 cycles of 3 min at 50˚C, with a final extension of 10 min at 72˚C. PCR products were separated by electrophoresis on 12% non-denatured polyacrylamide gels and stained by AgNO3 solution [49]. Polymorphic SRAP markers were scored as binary data with presence (1) or absence (0).

### Statistical analyses

The Kolmogorov–Smirnov and Bartlett’s tests were used to test the normal distribution of the data and homogeneity of variances, respectively. Subsequently, a combined analysis of variance, proposed by Steel and Torrie [50], was conducted using Proc MIXED of SAS release 9.4 (SAS Institute, Cary, NC, USA) to examine the differences between the genotypes, moisture regimes, and their interaction [51]. The least significant difference (LSD) test at P<0.05 was used for trait means comparison [50]. Data were also subjected to ANOVA separately for normal and water deficit regimes and variance components were estimated for individual moisture regimes using proc MIXED of SAS. Broad-sense heritability (h^2^b) was estimated for normal and water deficit regimes on a phenotypic mean basis averaged over replications as follows (Eq 4) [52]:

$$h^{2}=\sigma^{2}g/(\sigma^{2}g+\sigma^{2}gy/y+\sigma^{2}gr/r+\sigma^{2}e/ry)$$ (4)

where *σ*^*2*^*g* is the genotype, *σ*^*2*^*gy* is the genotype × year, *σ*^*2*^*rg* is the genotype × rep, and *σ*^*2*^*e* is the residual variance, y is the number of years and r is the number of replicates. To estimate the level of genetic variation, the genetic coefficient of variation (GCV) was calculated as:

GCV=(σg/μ)100 (5)

where σg is the standard deviation of the genotypic variance, and μ is the phenotypic mean [53].

### Population structure and association analysis

Structure analysis and stratification of the studied population into subpopulations with different genetic structures were done based on SRAP marker data in STRUCTURE version 2.3.4 [27]. This analysis applied an admixture model, a burn-in of 10,000 iterations followed by 100,000 Monte Carlo Markov Chain (MCMC) replicates. The membership of each genotype was run for the range of genetic clusters (K) from K = 2 to K = 10 with five repetitions for each K. Delta k approach by Evanno et al. [54] was used to determine the optimum number of sub-populations, using STRUCTURE HARVESTER [55].

Association analysis was run by MLM [28] to calculate *P*-values for marker–trait associations using TASSEL version 4.2.1 [56]. The phenotypic mean of traits (P-matrix) was applied to separately identify significant associations under normal and water deficit regimes. The Q-matrix derived from structure analysis (at maximum DK) was used as a covariate to correct for population structure in the MLM model. Moreover, a kinship matrix (K-matrix) was calculated based on the results of marker genotype data using TASSEL version 4.2.1 [56]. A correction for multiple testing was performed with the FDR (false discovery rate) method, using the QVALUE R package [57].

## Results

### Phenotyping

As stated earlier, before analysis of variance, normality test was performed for all of the studied traits using the Kolmogorov–Smirnov test and its results verified the normality of all data (S3 Table). The Bartlett test for homogeneity of variance also showed the homogeneity of variance for moisture regimes. Therefore, the data of these two environments were combined for the analysis of variance. Based on the results of ANOVA, significant differences were observed between the normal and water deficit regimes for all traits except for PER. The effect of genotype was significant for all measured traits, indicating significant variation among the selected genotypes with a broad range for each trait. Genotype × environment (GE) interaction was significant for all the evaluated traits except for PER (Table 1).

**Table 1:** Combined analysis of variance for measured traits in 36 genotypes of smooth bromegrass evaluated under two moisture regimes (normal and water deficit).

Source of variations	Degree of freedom	Mean squares								
		DMY1-Y1	DMY2-Y1	DMY1-Y2	DMY2-Y2	DMY1-Y3	DMY2-Y3	RY	DRAD	PER
Environment (E)	1	229389.65**	18910.09**	752476.57**	157045.72**	918523.85**	22384.64**	1446.27*	541.94**	1857.12^n.s^
Replication/E	10	12854.74**	583.00**	9794.84**	4813.23**	11104.06**	565.13**	3274.34**	22.14**	35682.63**
Genotype (Gen)	35	6033.27**	427.69**	10297.59**	1185.59**	9343.21**	425.53**	1577.63**	15.46**	16470.32**
Gen × E	35	2716.00**	227.04**	5659.22*	860.27**	4998.26**	326.79**	774.17**	7.06**	3899.02^n.s^
Error	350	1422.10	79.28	3507.00	356.84	2553.62	110.36	372.14	3.26	4046.13

** significant at the 0.01 probability level; ns: not significant

DMY1-Y1, dry matter yield of cut 1 in the first year; DMY2-Y1, dry matter yield of cut 2 in the first year; DMY1-Y2, dry matter yield of cut 1 in the second year; DMY2-Y2, dry matter yield of cut 2 in the second year; DMY1-Y3, dry matter yield of cut 1 in the third year; DMY2-Y3, dry matter yield of cut 2 in the third year; DRAD, degree of recovery after drought; PER, persistence; RY, recovery yield.

Mean comparisons of all measured traits for the two moisture regimes are given in Table 2. Results showed that the moisture regime had a significant effect on all of the evaluated traits, and the magnitude of mean performance was significantly decreased for all traits under water deficit regime (Table 2). Under water deficit, DMY1 was approximately reduced by 36, 39, and 37% during 2013, 2014, and 2015, respectively, compared with the normal regime. For DMY2, these reductions were approximately 38, 60, and 56% in the same three consecutive years relative to normal regime. Moreover, water deficit decreased the RY and PER of the genotypes by 35 and 28%, respectively.

**Table 2:** Mean performance, range (Min and Max), genotypic coefficient of variation (GCV) and broad- sense heritability (h2b) of traits recorded under normal and water deficit regimes in smooth bromegrass genotypes.

Traits	Mean ± SD		Change (%)	Range				GCV (%)		h^2^_b_ (%)		
				Min		Max						
	Normal	water deficit		Normal	water deficit	Normal	water deficit	Normal	water deficit	Combined	Normal	water deficit
DMY1-Y1 (g/plant)	155.66 ± 41.77	100.24 ± 33.96	35.60**	31.80 (G27)	31.00 (G27)	168.80 (G26)	136.40 (G8)	22.59	29.27	73.72	75.53	77.19
DMY2-Y1 (g/plant)	30.48 ± 6.36	18.81 ± 5.42	38.29**	10.60 (G27)	8.00 (G34)	55.17 (G33)	29.67 (G6)	27.35	24.18	75.93	77.61	70.50
DMY1-Y2 (g/plant)	264.59 ± 68.05	161.33 ± 46.50	39.03**	111.60 (G1)	70.20 (G2)	319.00 (G10)	205.75 (G3)	22.11	27.48	70.20	79.38	77.81
DMY2-Y2 (g/plant)	61.76 ± 17.19	24.81 ± 7.86	59.83**	17.33 (G1)	3.00 (G36)	96.00 (G4)	16.50 (G21)	22.50	26.80	60.53	66.39	72.05
DMY1-Y3 (g/plant)	155.56 ± 42.81	98.40 ± 28.16	36.74**	47.67 (G1)	10.33 (G11)	232.00 (G24)	84.83 (G7)	22.98	24.42	72.69	70.77	74.27
DMY2-Y3 (g/plant)	36.92 ± 9.31	16.29 ± 4.55	55.88**	9.67 (G20)	4.33 (G36)	63.50 (G24)	23.00 (G13)	19.87	22.26	59.58	60.99	63.34
RY (g/plant)	39.77 ± 11.10	25.90 ± 16.75	34.87 **	9.13 (G3)	3.50 (G15)	50.70 (G28)	63.40 (G32)	27.14	55.10	50.93	52.97	75.47
DRAD (0–9)	5.45 ± 1.08	3.19 ± 1.62	41.47**	3.17 (G21)	1.00 (G15)	7.67 (G9)	6.67 (G7)	14.71	44.66	54.37	54.79	78.46
PER (g/plant)	69.27 ± 29.80	49.66 ± 14.35	28.31 **	46.59 (G18)	30.47 (G36)	169.70 (G29)	115.4 (G30)	68.15	52.49	76.33	61.03	58.78
SDI-Y1	8.60 ± 0.34	—	—	4.54 (G17)	—	8.97 (G8)	—	2.86	—	—	56.25	—
SDI-Y2	7.61 ± 0.56	—	—	6.17 (G17)	—	8.67 (G14)	—	5.86	—	—	64.57	—
SDI-Y3	7.44 ± 0.74	—	—	6.16 (G4)	—	8.86 (G19)	—	7.96	—	—	71.76	—

** significant at the 0.01 probability level; ns: not significant

DMY1-Y1, dry matter yield of cut 1 in the first year; DMY2-Y1, dry matter yield of cut 2 in the first year; DMY1-Y2, dry matter yield of cut 1 in the second year; DMY2-Y2, dry matter yield of cut 2 in the second year; DMY1-Y3, dry matter yield of cut 1 in the third year; DMY2-Y3, dry matter yield of cut 2 in the third year; DRAD, degree of recovery after drought; PER, persistence; RY, recovery yield; SDI-Y1, summer dormancy index of the first year; SDI-Y2, summer dormancy index of the second year; SDI-Y3, summer dormancy index of the third year.

In the fourth year (2016), after withholding irrigation in both moisture regimes and re-watering, no mortality of smooth bromegrass genotypes was observed and all recovered. Mean comparisons of recovery-related traits indicated that genotypes 9 and 28 had higher values of recovery vigor in normal regime, and genotypes 7 and 32 had the higher values under water deficit. The lowest recovery vigor was observed in genotypes 3 and 21 under normal regime and in genotype 15 under water deficit (Table 2). Moreover, the magnitude of persistence varied significantly among the genotypes under both moisture regimes. Under normal regime, the highest value of PER was observed for G29, and the lowest value was detected for G18. Genotypes 30 and 36 had the highest and lowest values of PER under water deficit regime, respectively (Table 2).

Genotypes of this germplasm showed incomplete summer dormancy; therefore, the SDI was calculated as the ratio of a genotype’s summer yield to the same genotype’s spring yield. Using the SDI based on summer forage production under normal regime, genotypes 4 and 17 were scored the least dormant. While genotypes 8, 14, and 19 showed the higher summer dormancy (Table 2).

In the present study, the genetic coefficient of variation (GCV) was from 2.86% for SDI-Y1 to 68.15% for PER in normal regime and from 22.26% for DMY2-Y3 to 55.10% for RY under water deficit regime. Except for DMY2-Y1 and PER, the values of genetic variation under water deficit were higher than the ones for normal regime (Table 2).

Broad-sense heritability estimates for combined data ranged from 50.93% for RY to 76.33% for PER. Moreover, the heritability estimates were calculated for each moisture regime separately and are given in Table 2. Under both moisture regimes, moderate to high heritability values were estimated for all traits. According to the results, heritability estimates ranged from 52.97% for RY to 79.38% for DMY1-Y2 in normal regime and from 58.78% for PER to 78.46% for DRAD under water deficit regime (Table 2).

### Population structure and association analysis

**Fig 1:**
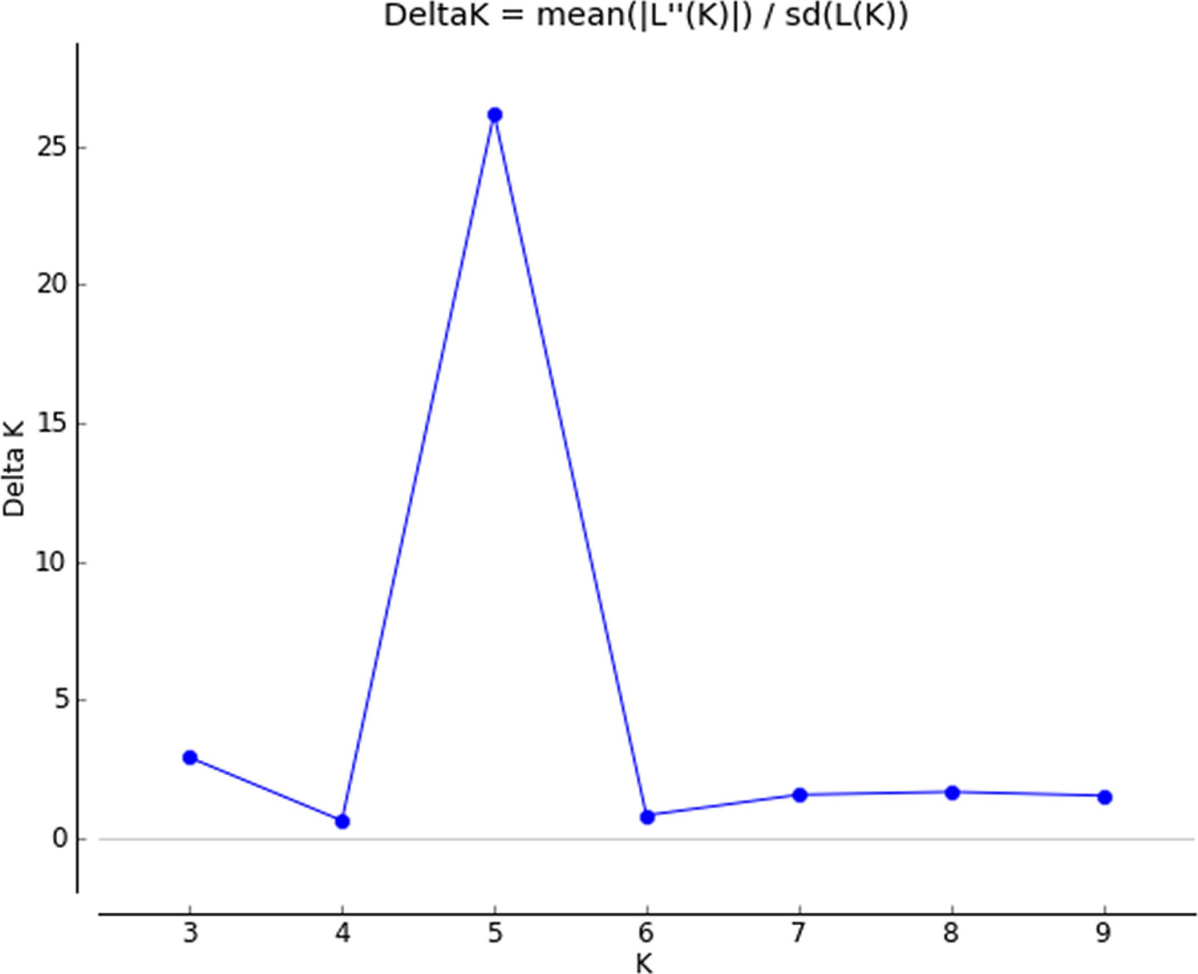
Population structure analysis in a diverse panel of smooth bromegrass genotypes (Δk was used to determine the optimum K value for population structure using the Bayesian clustering method).

**Fig 2:**
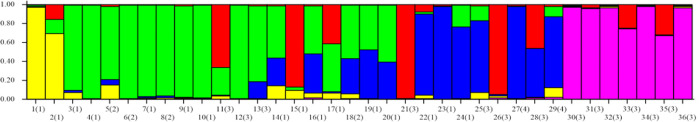
Genetic relatedness of smooth bromegrass genotypes analyzed by STRUCTURE program. Numbers on the *y*-axis indicate the membership coefficient. The color of the bar indicates the five sub-groups identified through the STRUCTURE program. Genotypes with the same color belong to the same group.

The maximum likelihood and D*K* were used to calculate the number of subpopulations (*K*). The optimum number of sub-populations (K) was determined based on 30 SRAP primer combinations using the largest value of Delta K in the STRUCTURE 2.3.4 software. The maximum value of D*K* was obtained at *K* = 5 suggesting that there were five subpopulations in the smooth bromegrass panel (S4 Table; Figs 1 and 2).

The association analysis between SRAP markers and the phenotypic mean of traits was conducted separately for normal and water deficit regimes based on the MLM model. Under the normal regime (*P* values <0.01 and a cut-off value of 0.05 for the FDR), 69 SRAP markers showed significant associations with means of the studied traits at the 0.01 probability level (Table 3). The percentage of phenotypic variation (coefficient of determination, *R*^2^) range was from 2.72% to 11.38% (Table 3). Under water deficit, 46 markers were significantly associated with the studied traits at the 0.01 probability level. The percentage of phenotypic variation (*R*^2^) range was variable from 1.77% to 16.03% (Table 3). It should be stated that, from SRAP markers related to studied traits under normal regime, 21 were significantly associated with SDI-Y1, SDI-Y2, and SDI-Y3 (which were only calculated under normal regime), at the 0.01 probability level (Table 3).

**Table 3:** Association of SRAP markers with dry matter yield, persistence, summer dormancy, and recovery traits of smooth bromegrass under normal and water deficit regimes based on mixed linear model (MLM).

Traits	Normal regime			Water deficit regime		
	Marker	P value	R^2^ (%)	Marker	P value	R^2^ (%)
DMY1-Y1	Me5/Em4-7 ^†^	0.0005	5.44	Me2/Em4-16	0.00023	6.35
	Me1/Em4-19 ^†^	0.0029	4.20	Me2/Em1-1	0.0021	4.71
	Me1/Em2-15	0.0032	4.13	Me1/Em4-19 ^†^	0.0029	4.47
	Me4/Em3-11	0.0039	3.98	Me5/Em4-7 ^†^	0.0038	4.25
				Me4/Em1-16	0.0068	3.78
DMY2-Y1	Me2/Em2-16	0.002	4.86	Me2/Em4-16	0.0007	7.56
	Me4/Em3-11	0.002	4.84	Me5/Em2-13	0.0022	6.40
	Me4/Em4-21 ^†^	0.0024	4.70	Me1/Em1-17	0.0032	6.00
	Me2/Em3-4	0.0025	4.66	Me4/Em1-16	0.0047	5.57
	Me1/Em2-20	0.0035	4.40	Me5/Em3-23	0.0067	5.18
	Me5/Em5-6	0.0044	4.21	Me4/Em4-21 ^†^	0.0086	4.90
	Me4/Em6-4	0.0058	3.99			
	Me2/Em5-7	0.007	3.82			
DMY1-Y2	Me5/Em4-4	0.0034	3.32	Me1/Em1-17	0.00034	4.81
	Me4/Em2-12	0.0041	3.20	Me2/Em4-16	0.0054	3.15
	Me1/Em3-15	0.0086	2.74			
	Me5/Em3-1	0.0089	2.72			
DMY2-Y2	Me1/Em5-11 ^†^	0.0014	6.95	Me5/Em2-20	0.0032	5.03
	Me1/Em3-15	0.0028	6.18	Me5/Em3-17	0.0058	4.47
	Me4/Em2-12	0.0058	5.39	Me1/Em4-8	0.0061	4.42
	Me1/Em2-1	0.0074	5.12	Me5/Em4-10	0.0064	4.38
	Me5/Em3-4	0.0087	4.93	Me1/Em5-11 ^†^	0.0075	4.22
	Me4/Em3-11	0.0089	4.90			
	Me4/Em6-20	0.0093	4.86			
DMY1-Y3	Me2/Em5-12	0.0006	5.88	Me2/Em4-12 ^†^	0.0036	3.93
	Me5/Em5-19	0.0036	4.45	Me5/Em2-20	0.0037	3.92
	Me1/Em1-17 ^†^	0.0039	4.39	Me1/Em5-11	0.0068	3.46
	Me5/Em4-11	0.0056	4.09	Me1/Em1-17 ^†^	0.0085	3.30
	Me2/Em4-12 ^†^	0.0069	3.91			
	Me2/Em6-18	0.0083	3.76			
	Me1/Em2-6	0.0084	3.75			
	Me1/Em3-2	0.0091	3.68			
	Me5/Em2-15	0.0093	3.66			
DMY2-Y3	Me1/Em2-1	0.0016	8.88	Me5/Em4-4 ^†^	0.000558	9.36
	Me5/Em4-4 ^†^	0.0065	6.86	Me1/Em5-11	0.000997	8.66
	Me4/Em2-12	0.007	6.76	Me4/Em6-10	0.0021	7.74
	Me2/Em3-4	0.0094	6.33	Me1/Em3-19	0.0033	7.13
				Me1/Em3-15	0.0078	6.01
				Me4/Em1-13	0.0081	5.95
				Me5/Em5-18	0.0082	5.94
RY	Me4/Em2-12	0.0015	11.17	Me2/Em4-12	0.0041	3.13
	Me2/Em5-2	0.0024	10.41	Me1/Em1-1	0.0068	2.83
	Me1/Em3-19	0.005	9.07	Me4/Em6-12	0.0071	2.80
				Me1/Em3-15	0.0072	2.79
				Me1/Em4-15	0.0074	2.78
				Me1/Em4-7	0.0086	2.68
				Me5/Em3-5	0.0092	2.64
DRAD	Me1/Em4-9	0.000294	9.82	Me4/Em6-12	0.0025	2.25
	Me1/Em3-19	0.000633	8.95	Me2/Em4-12	0.0085	1.77
	Me4/Em4-15	0.0081	5.79			
PER	Me5/Em5-19 †	0.0015	8.63	Me4/Em1-16	0.000013	16.03
	Me5/Em2-3	0.0033	7.57	Me2/Em2-17	0.000975	10.40
	Me4/Em2-19	0.0044	7.18	Me2/Em1-2	0.0019	9.42
	Me2/Em3-13	0.005	7.01	Me1/Em1-7	0.0025	9.00
	Me2/Em1-13	0.0056	6.83	Me5/Em5-19 †	0.0058	7.67
	Me5/Em6-16	0.0075	6.43	Me5/Em4-10	0.0062	7.57
				Me1/Em3-18	0.0066	7.46
				Me5/Em2-13	0.0084	7.08
SDI-Y1	Me5/Em4-12	0.0000935	11.38			
	Me4/Em5-5	0.000993	8.66			
	Me2/Em3-22	0.0012	8.41			
	Me2/Em1-13	0.004	6.89			
	Me4/Em4-19	0.0051	6.59			
	Me1/Em5-24	0.0071	6.13			
	Me1/Em2-17	0.0075	6.06			
	Me1/Em3-21	0.0075	6.06			
SDI-Y2	Me5/Em1-8	0.0049	3.87			
	Me2/Em4-20	0.0052	3.83			
	Me5/Em3-18	0.0097	3.33			
SDI-Y3	Me5/Em3-6	0.00006	8.61			
	Me1/Em2-20	0.00009	8.34			
	Me4/Em6-4	0.00079	6.53			
	Me4/Em2-4	0.0018	5.80			
	Me4/Em5-5	0.0021	5.63			
	Me1/Em1-20	0.0022	5.62			
	Me1/Em6-21	0.0066	4.56			
	Me2/Em5-8	0.0088	4.27			
	Me4/Em2-25	0.0092	4.23			
	Me5/Em2-3	0.0095	4.20			

† Stable markers under normal and water deficit regimes.

DMY1-Y1, dry matter yield of cut 1 in the first year; DMY2-Y1, dry matter yield of cut 2 in the first year; DMY1-Y2, dry matter yield of cut 1 in the second year; DMY2-Y2, dry matter yield of cut 2 in the second year; DMY1-Y3, dry matter yield of cut 1 in the third year; DMY2-Y3, dry matter yield of cut 2 in the third year; DRAD, degree of recovery after drought; PER, persistence; RY, recovery yield; SDI-Y1, summer dormancy index of the first year; SDI-Y2, summer dormancy index of the second year; SDI-Y3, summer dormancy index of the third year.

Association analysis showed that several SRAP markers were associated with more than one trait. Under normal regime, 13 markers and under water deficit, ten markers showed significant associations with more than one trait, simultaneously. For example, under normal regime, marker Me4/Em2-12 showed significant associations with DMY1-Y2, DMY2-Y2, DMY2-Y3, and RY, and marker Me4/Em3-11 had significant associations with DMY1-Y1, DMY2-Y1, and DMY2-Y2, concurrently. In addition, under water deficit, marker Me1/Em1-17 showed significant associations with DMY2-Y1, DMY1-Y2, and DMY1-Y3, and marker Me1/Em5-11 had significant associations with DMY2-Y2, DMY1-Y3, and DMY2-Y3 (Table 3).

Association analysis was performed in each moisture regime separately to assess stable associations. In total, eight trait associated markers showed sufficiently stable expression across moisture regimes. For instance, markers Me5/Em4-7 and Me1/Em4-19 showed significant and stable associations with DMY1-Y1 in both moisture regimes. Similarly, Me1/Em1-17 and Me2/Em4-12 showed significant and stable associations with DMY1-Y3 (Table 3 and S5 Table).

An analysis was conducted on the traits of each experimental year to assess the stability of marker-trait associations (MTAs) between years. Results revealed five and three stable MTAs between years, under normal and water deficit regimes, respectively (Table 3 and S5 Table). For example, under normal regime Me4/Em2-12 and Me1/Em2-1 showed significant associations with DMY2-Y2 and DMY2-Y3. Similarly, marker Me4/Em5-5 had significant and stable associations with SDI-Y1 and SDI-Y3 under normal regime. On the other hand, Me2/Em4-16 showed significant and stable associations with DMY1-Y1 and DMY1-Y2, under water deficit regime.

## Discussion

The quantitative inheritance of drought tolerance and interaction between gene expression and environment has challenged the understanding of the genetic basis of drought tolerance-related traits in plants [34]. In the present study, significant genetic variations were observed among genotypes in terms of all measured traits demonstrating the difference in genes controlling these traits. Results also revealed the non-static performance of genotypes in the two moisture regimes. This was also evident by the expression of different MTAs in the two moisture regimes for the evaluated traits. The non-static performance of genotypes in the two moisture regimes emphasizes the importance of marker-trait association analysis in each moisture regime, separately.

All traits were significantly affected by water deficit, more likely due to decreased water potential of the soil and decline in net assimilation and photosynthesis of leaves [58]. Shariatipour et al. [59] in Kentucky bluegrass (*Poa pratensis* L.), Saeidnia et al. [7] in smooth bromegrass (*Bromus inermis*), and Majidi et al. [60] in orchardgrass (*Dactylis glomerata*) reported similar results. Wide genetic variation observed for all studied traits revealed the potential for genetic gain from selection in this germplasm. Moreover, higher estimates of GCV for most of the evaluated traits under water deficit than non-stress indicate that water deficit has increased genetic variation. Therefore, selection under this condition would be more effective. The findings in this context are contradictory. For example, some researchers believe that genetic gain through selection is higher in normal regime than water-limited conditions [60, 61]. At the same time, others have reported higher genetic advances through selection under water deficit [62]. Moderate to high heritability estimates observed for all traits emphasize that detecting marker–trait associations are possible for these traits [63].

In the present study, mortality of smooth bromegrass plants was not observed due to withholding irrigation. Meanwhile, Abdollahi et al. [64] in orchardgrass and Pirnajmedin et al. [65] in tall fescue (*Festuca arundinacea* Schreb.) reported that, after withholding irrigation, some of the genotypes died under both well-watered and deficit irrigation regimes. The superiority of smooth bromegrass in this respect may be related to greater rhizomes in length and volume, which would serve as soluble carbohydrate storage organs during stress [66]. Moreover, high genetic variation was found among the genotypes for recovery-related traits (RY and DRAD), indicating good potential for genetic study of these traits and the possibility of selection of genotypes with variable recovery and survival vigor in this germplasm collection. Results of the recovery experiment also revealed that both recovery-related traits declined under water deficit compared with non-stress environment. Because the water deficit was applied identically in all three years before withholding irrigation, it can be concluded that prolonged water deficit reduced the persistence and recovery of plants [7]. It seems that the reduction of persistence is possibly related to the decreased crown diameter, lesser root growth, and potentially, a decrease in growing points, which results in decreasing plant vigor season-over-season. The persistence of perennial grasses is dependent on maintaining strong tiller stocks. Populations decline if tillers are not replaced in the following seasons. Therefore, population survival and persistence are strongly indicated by tiller density [67].

In this study, summer forage yields were significantly lower than the spring ones under normal and water-deficit regimes. Therefore, genotypes of this germplasm showed low to moderate summer dormancy. Norton et al. [18] and Saeidnia et al. [42] found similar results in orchardgrass and smooth bromegrass, respectively. In arid regions, it is preferred to select plant types with incomplete summer dormancy and higher drought tolerance, as this is associated with better yearly production.

Association analysis of evaluated traits under normal and water deficit regimes revealed that MTAs were mostly different in the two moisture regimes. Moreover, results showed that more genes were involved in controlling traits under normal regime than in water deficit. The percentage of variation explained by each identified association was low. This may be explained by the role of many minor genes controlling the trait, outcrossing nature of smooth bromegrass, markers exhibiting minor quantitative effects, rare alleles, and complex allelic interactions [68, 69]. Similar results were reported by Lou et al. [33] and Sun et al. [34] in tall fescue.

Based on the results, some markers showed significant associations with more than one trait which would be attributed to the pleiotropic effects or several tightly linked genes affecting these traits [34, 70]. These markers may be effectively used to improve several traits concurrently [34, 37]. Association between multiple traits could be attributed to the co-expression mediated by quantitative trait loci (e-QTLs) [71].

Improving the persistence, recovery, and survivability of perennial forage grasses is one of the main objectives of grass breeders in areas with prolonged periods of drought [4, 5]. This study identified markers associated with these traits under normal and water-deficit regimes. Suppose the effectiveness of these regions in the genetic control of these traits is confirmed. In that case, these markers could be potentially used to improve persistence and drought tolerance of smooth bromegrass. Moreover, an important trait associated with survivability, persistence, and recovery after the drought is summer dormancy, which improves autumn recovery and therefore results in a better persistence of perennial grasses. In the present study, eight, three, and ten markers were associated with SDI-Y1, SDI-Y2, and SDI-Y3, respectively. From these markers, four were also associated with other traits and one marker (Me4/Em5-5) showed a stable association with SDI at two years of study.

In this study, most of the MTAs were different for the two moisture regimes and the three experimental years, indicating the considerable role of environmental effects in these associations [72]. These findings suggest that different genes contribute to the same trait in different environments and years or the same genes may change the expression level between different environments [71]. In the present study, eight markers showed stable association with different traits under both moisture regimes. Moreover, five and three markers showed stable MTAs between two or three years of study, under normal and water deficit regimes, respectively. In general, associated markers detected in two or more different environments or experimental years are more reliable than those present in only one environment [73]. However, these SRAP–trait associations must be validated in independent germplasm for use in marker-assisted selection programs.

## Conclusions

In conclusion, the advantage of the association analysis technique as a powerful tool to identify and detect genes and markers linked to complex traits of agricultural and economic importance was demonstrated. Satisfactory levels of polymorphism and genetic diversity were observed for the studied traits in the polycrossed population. Five subpopulations were identified in the smooth bromegrass panel, and 69 and 46 significant MTAs were detected under normal and water deficit regimes, respectively. Environmental specificity of MTAs indicated that genotype × environment interactions affect association analysis; nevertheless, eight markers showed stable association with different traits across moisture regimes; and five and three markers showed significantly stable expression across two or three years of study, under normal and water deficit regimes, respectively. However, most of the MTAs were different for the two moisture regimes and the three experimental years, indicating the considerable role of environmental effects in these associations. This suggests that different genes contribute to the same trait in different environments and years or the same genes may change the expression level between different environments. Based on the results, some markers showed significant associations with more than one trait demonstrating reliability of these markers for marker-assisted selection (MAS) of correlated traits concurrently. The molecular markers identified in the present study are suggested as valuable genomic resources in the future breeding programs of smooth bromegrass. However, these SRAP–trait associations must be validated in independent germplasm for use in marker-assisted selection programs.

## Supporting information

S1 Table Information on parental plants of genetic materials used in this study. (DOC)

S2 Table Abbreviation, description, and unit of measurement for the evaluated traits of smooth bromegrass in this study. (DOC)

S3 Table Results of Kolmogorov-Smirnov test for normality of data. The p-value larger than 0.05 indicates that the data follow normal distribution.
(DOC)

S4 Table Calculated statistics to detect optimum number of subgroups (*K*) in structure analysis of smooth bromegrass genotypes (D*K* method; Evanno *et al*. 2005), using the STRUCTURE HARVESTER. (DOC)

S5 Table Stable markers during years and moisture regimes (normal and water deficit) for evaluated traits. (DOC)

S1 Raw data SRAP data. (XLSX)

S2 Raw data Phenotypic data under normal and water deficit regimes. (XLSX)
