# Supplementary material for: Association analysis revealed loci linked to post-drought recovery and traits related to persistence of smooth bromegrass (Bromus inermis)
Source: PLoS One. 2022 Dec 7;17(12):e0278687. doi: 10.1371/journal.pone.0278687 (PMC9728867; doi:10.1371/journal.pone.0278687)
Supplement: S1 Table — (DOC) [file pone.0278687.s001.doc]

| **S1 Table-** Information on parental plants of genetic materials used in this study. | | |
| --- | --- | --- |
| Parental plant | Population code | Origin |
| 1 | 2000/25 | Iran, Hamedan |
| 2 | 2000/18 | Iran, Isfahan-Fozve |
| 3 | 2000/50 | Iran, Isfahan-Fozve |
| 4 | 2000/40 | Iran, Isfahan-Semirom |
| 5 | 2000/43 | Iran, Isfahan-Fozve |
| 6 | 2000/4 | Iran, Isfahan-Fozve |
| 7 | 2000/18-2 | Iran, Isfahan-Fozve |
| 8 | 2000/T-9 | Iran, Hamedan |
| 9 | 2000/60 | Iran, Semnan |
| 10 | 2000/10 | Iran, Kordestan |
| 11 | 2000/24 | Iran, Isfahan-Fozve |
| 12 | RCAT040601 | Hungary |
| 13 | RCAT041016 | Hungary |
| 14 | RCAT041861 | Hungary |
| 15 | RCAT042133 | Hungary |
| 16 | RCAT042134 | Hungary |
| 17 | RCAT064831 | Hungary |
| 18 | RCAT064835 | Hungary |
| 19 | RCAT064837 | Hungary |
| 20 | RCAT064839 | Hungary |
| 21 | - | Iran, Isfahan-Fozve |
| 22 | - | Iran, Isfahan-Fozve |
| 23 | - | Iran, Isfahan-Fozve |
| 24 | - | Iran, Isfahan-Fozve |
| 25 | - | Iran, Isfahan-Fozve |
