# Supplementary material for: Association analysis revealed loci linked to post-drought recovery and traits related to persistence of smooth bromegrass (Bromus inermis)
Source: PLoS One. 2022 Dec 7;17(12):e0278687. doi: 10.1371/journal.pone.0278687 (PMC9728867; doi:10.1371/journal.pone.0278687)
Supplement: S2 Table — (DOC) [file pone.0278687.s002.doc]

| **S2 Table-** Abbreviation, description, and unit of measurement for the evaluated traits of smooth bromegrass in this study. | | | |
| --- | --- | --- | --- |
| **Characters** | **Abbreviation** | **Unit** | **Description** |
| Dry matter yield of cut 1 in year 1 | DMY1-Y1 | (g/plant) | The weight of the aboveground biomass (foliage) of each plant after drying in 75˚C for 48 h at the first cut of the first year |
| Dry matter yield of cut 2 in year 1 | DMY2-Y1 | (g/plant) | The weight of the aboveground biomass (foliage) of each plant after drying in 75˚C for 48 h at the second cut of the first year |
| Dry matter yield of cut 1 in year 2 | DMY1-Y2 | (g/plant) | The weight of the aboveground biomass (foliage) of each plant after drying in 75˚C for 48 h at the first cut of the second year |
| Dry matter yield of cut 2 in year 2 | DMY2-Y2 | (g/plant) | The weight of the aboveground biomass (foliage) of each plant after drying in 75˚C for 48 h at the second cut of the second year |
| Dry matter yield of cut 1 in year 3 | DMY1-Y3 | (g/plant) | The weight of the aboveground biomass (foliage) of each plant after drying in 75˚C for 48 h at the first cut of the third year |
| Dry matter yield of cut 2 in year 3 | DMY2-Y3 | (g/plant) | The weight of the aboveground biomass (foliage) of each plant after drying in 75˚C for 48 h at the second cut of the third year |
| Recovery yield | RY | (g/plant) | The dry weight of the above-ground biomass of each genotype after withholding irrigation and re-watering |
| Degree of recovery after drought | DRAD | - | The visually score of the leaves of each plant based on a scale of 0-9 (green and fully hydrated leaves: 9; desiccated brown or dead leaves:0) |
| Persistence | PER | (g/plant) | The difference in dry matter yield of the first cut in the fourth year (2016) from the dry matter yield of the first cut in the second year (2014) |
| Summer dormancy index in year 1 | SDI-Y1 | - | the ratio of the DMY1 (SUFY) of a genotype at the first year to the DMY2 (SPFY) of the same genotype at the first year |
| Summer dormancy index in year 2 | SDI-Y2 | - | the ratio of the DMY1 (SUFY) of a genotype at the second year to the DMY2 (SPFY) of the same genotype at the second year |
| Summer dormancy index in year 3 | SDI-Y3 | - | the ratio of the DMY1 (SUFY) of a genotype at the third year to the DMY2 (SPFY) of the same genotype at the third year |
