# Supplementary material for: Association analysis revealed loci linked to post-drought recovery and traits related to persistence of smooth bromegrass (Bromus inermis)
Source: PLoS One. 2022 Dec 7;17(12):e0278687. doi: 10.1371/journal.pone.0278687 (PMC9728867; doi:10.1371/journal.pone.0278687)
Supplement: S3 Table — The p-value larger than 0.05 indicates that the data follow normal distribution. (DOC) [file pone.0278687.s003.doc]

| **S3 Table -** Results of Kolmogorov-Smirnov test for normality of data. The p-value larger than 0.05 indicates that the data follow normal distribution. | | |
| --- | --- | --- |
| Traits | Statistic | P Value |
| DMY1-Y1 (g/plant) | 0.118454 | 0.15 |
| DMY2-Y1 (g/plant) | 0.124125 | 0.15 |
| DMY1-Y2 (g/plant) | 0.131709 | 0.1130 |
| DMY2-Y2 (g/plant) | 0.095634 | 0.15 |
| DMY1-Y3 (g/plant) | 0.098012 | 0.15 |
| DMY2-Y3 (g/plant) | 0.192161 | 0.10 |
| RY (g/plant) | 0.093795 | 0.15 |
| DRAD (0-9) | 0.115516 | 0.15 |
| PER (g/plant) | 0.099791 | 0.15 |
| SDI-Y1 | 0.109497 | 0.15 |
| SDI-Y2 | 0.087247 | 0.15 |
| SDI-Y3 | 0.127085 | 0.1452 |
