# Supplementary material for: Association analysis revealed loci linked to post-drought recovery and traits related to persistence of smooth bromegrass (Bromus inermis)
Source: PLoS One. 2022 Dec 7;17(12):e0278687. doi: 10.1371/journal.pone.0278687 (PMC9728867; doi:10.1371/journal.pone.0278687)
Supplement: S4 Table — (DOC) [file pone.0278687.s004.doc]

| **S4 Table-** Calculated statistics to detect optimum number of subgroups (*K*) in structure analysis of smooth brome genotypes (D*K* method; Evanno *et al*. 2005), using the program STRUCTURE. | | | | | | |
| --- | --- | --- | --- | --- | --- | --- |
| K | Reps | Mean LnP (K) | Stdev LnP (K) | Ln′ (K) | Ln′′ (K) | ΔK |
| 2 | 5 | -16431.74 | 7.34 | — | — | — |
| 3 | 5 | -16407.62 | 33.96 | 24.12 | 98.10 | 2.89 |
| 4 | 5 | -16481.60 | 170.23 | -73.98 | 103.00 | 0.61 |
| 5 | 5 | -16658.58 | 341.65 | -176.98 | 8949.18 | 26.19 |
| 6 | 5 | -25784.74 | 11478.06 | -9126.16 | 9152.34 | 0.80 |
| 7 | 5 | -25758.56 | 5338.22 | 26.18 | 8224.16 | 1.54 |
| 8 | 5 | -33956.54 | 6821.92 | -8197.98 | 11231.70 | 1.65 |
| 9 | 5 | -30922.82 | 10902.48 | 3033.72 | 16460.40 | 1.51 |
| 10 | 5 | -44349.50 | 18473.15 | -13426.68 | — | — |
| Mean LnP (*K*), mean of LnP(D) of repetitions for each *K*; Stdev LnP (*K*), standard deviation of repetitions; Ln′ (*K*), Ln (*K*)*n* – Ln (*K*)*n* – 1; Ln′′ (*K*), Ln′ (*K*)*n* – Ln′ (*K*)*n* – 1; D*K*, |Ln′′ (*K*)|/stdev LnP (*K*). *, *K*-value with largest D*K*. | | | | | | |
