# Supplementary material for: Association analysis revealed loci linked to post-drought recovery and traits related to persistence of smooth bromegrass (Bromus inermis)
Source: PLoS One. 2022 Dec 7;17(12):e0278687. doi: 10.1371/journal.pone.0278687 (PMC9728867; doi:10.1371/journal.pone.0278687)
Supplement: S5 Table — (DOC) [file pone.0278687.s005.doc]

| **S5 Table-** Stable markers during years and moisture environments (normal and water stress) for evaluated traits. | | | | |
| --- | --- | --- | --- | --- |
| Traits | Stable markers during years | |  | Common markers between environments |
|  | Normal environment | Water stress environment |  |  |
| DMY1-Y1 | - | Me2/Em4-16 |  | Me5/Em4-7; Me1/Em4-19 |
| DMY2-Y1 | Me4/Em3-11; Me2/Em3-4 | - |  | Me4/Em4-21 |
| DMY1-Y2 | - | Me2/Em4-16; Me1/Em1-17 |  | - |
| DMY2-Y2 | Me4/Em3-11; Me4/Em2-12; Me1/Em2-1 | Me1/Em5-11 |  | Me1/Em5-11 |
| DMY1-Y3 | - | Me1/Em1-17 |  | Me1/Em1-17; Me2/Em4-12 |
| DMY2-Y3 | Me2/Em3-4; Me4/Em2-12; Me1/Em2-1 | Me1/Em5-11 |  | Me5/Em4-4 |
| RY | - | - |  | - |
| DRAD | - | - |  | - |
| PER | - | - |  | Me5/Em5-19 |
| SDI-Y1 | Me4/Em5-5 | - |  | - |
| SDI-Y2 | - | - |  | - |
| SDI-Y3 | Me4/Em5-5 | - |  | - |
| DMY1-Y1, dry matter yield of cut 1 in the first year; DMY2-Y1, dry matter yield of cut 2 in the first year; DMY1-Y2, dry matter yield of cut 1 in the second year; DMY2-Y2, dry matter yield of cut 2 in the second year; DMY1-Y3, dry matter yield of cut 1 in the third year; DMY2-Y3, dry matter yield of cut 2 in the third year; DRAD, degree of recovery after drought; PER, persistence; RY, recovery yield; SDI-Y1, summer dormancy index of first year; SDI-Y2, summer dormancy index of second year; SDI-Y3, summer dormancy index of third year. | | | | |
